# Complete Multipartite Genome Sequence of the Cupriavidus basilensis Type Strain, a 2,6-Dichlorophenol-Degrading Bacterium

**DOI:** 10.1128/MRA.00134-21

**Published:** 2021-05-13

**Authors:** Francisco Salvà-Serra, Raúl A. Donoso, Kyoung Hee Cho, Ji A Yoo, Kihyun Lee, Seok-Hwan Yoon, Beatriz Piñeiro-Iglesias, Edward R. B. Moore, Danilo Pérez-Pantoja

**Affiliations:** a Culture Collection University of Gothenburg (CCUG), Sahlgrenska Academy, University of Gothenburg, Gothenburg, Sweden; b Department of Infectious Diseases, Institute for Biomedicine, Sahlgrenska Academy, University of Gothenburg, Gothenburg, Sweden; c Microbiology, Department of Biology, University of the Balearic Islands, Palma de Mallorca, Spain; d Programa Institucional de Fomento a la Investigación, Desarrollo e Innovación, Universidad Tecnológica Metropolitana, Santiago, Chile; e Center of Applied Ecology and Sustainability (CAPES), Santiago, Chile; f ChunLab, Inc., Seoul, South Korea; Indiana University, Bloomington

## Abstract

We report the complete 8.94-Mb genome sequence of the type strain of Cupriavidus basilensis (DSM 11853 = CCUG 49340 = RK1), formed by two chromosomes and six putative plasmids, which offers insights into its chloroaromatic-biodegrading capabilities.

## ANNOUNCEMENT

The complete genome sequence of the type strain of Cupriavidus basilensis (← Wautersia basilensis ← Ralstonia basilensis ← *Ralstonia* sp.) (1–4) has been determined. Strain RK1^T^ (= DSM 11853^T^ = CCUG 49340^T^) was isolated from sediment from a freshwater pond in Amponville, France, with 2,6-dichlorophenol as the sole carbon and energy source (1).

Strain DSM 11853^T^ was cultivated on Reasoner’s 2A (R2A) broth, at 30°C, for 48 h. Genomic DNA was isolated, using a GenElute bacterial genomic DNA kit (Sigma-Aldrich) and a Wizard genomic DNA purification kit (Promega) for Illumina sequencing and a previously described protocol (5) for Oxford Nanopore sequencing. A DNA library was prepared, using a Nextera XT kit (Illumina) and sequenced on an Illumina HiSeq platform at MicrobesNG (Birmingham, UK), generating 3,305,358 paired-end reads of 251 bp. Another library was prepared, using a TruSeq Nano DNA sample preparation kit (Illumina), and sequenced on an Illumina MiSeq platform at ChunLab, Inc. (Seoul, South Korea), resulting in 4,445,298 paired-end reads of an average length of 292 bp. The reads were trimmed using Sickle v1.33 (Phred quality cutoff, Q30) (6) and assessed using CLC Genomics Workbench v12.0.3 (Qiagen).

Two Oxford Nanopore libraries were prepared, using a rapid barcoding sequencing kit (SQK-RBK004), and sequenced on a MinION device (Oxford Nanopore). The Nanopore reads were base called, using Guppy v2.3.7 and v3.1.5 (Oxford Nanopore) and evaluated, using NanoPlot v1.26.3 (7). The sequencing runs yielded 1.82 and 1.72 Gb, distributed in 291,236 and 243,691 reads, with *N*_50_ values of 11,574 and 12,956 bp, respectively.

The Illumina and Nanopore reads were assembled *de novo* using Unicycler v0.4.7 (8), resulting in complete circular sequences for all replicons except for chromosome 1, which was completed by assembling all Nanopore reads *de novo*, using Canu v1.5 (9). Subsequently, the sequence was polished with Illumina reads, using the tool Polish with Reads in CLC Genomics Workbench v20 (one round) and Pilon v1.20 (10) (two rounds). For Pilon, the reads were mapped using BWA v0.7.17 (11). The assembly statistics were obtained, using QUAST v5.0.2 (12). The complete genome sequence is composed of eight circular replicons, two chromosomes, and six putative plasmids, totaling 8,942,610 bp (Table 1). The sequence was annotated, using PGAP v4.13 (13) and BlastKOALA v2.2 (14), revealing 8,060 coding sequences (including 1,082 hypothetical proteins), 7 ribosomal operons, 67 tRNAs, and 236 pseudogenes, with a G+C content of 65.0 mol%.

**TABLE 1:** General features of the eight replicons of the complete genome sequence of *C. basilensis* DSM 11853^T^ (= CCUG 49340^T^ = RK1^T^)

Replicon	GenBank accession no.	Length (bp)	G+C content (mol%)	No. of CDS^*a*^	No. of ribosomal RNAs	No. of operons	No. of tRNAs	No. of hypothetical proteins (percentage of CDS)^*a*^
Chromosome 1	CP062803	4,566,734	65.3	4,123	12	4	54	464 (11)
Chromosome 2	CP062804	3,303,026	65.8	2,908	9	3	12	357 (12)
Plasmid pRK1-1	CP062805	425,364	61.0	387	0	0	1	90 (23)
Plasmid pRK1-2	CP062806	355,033	62.0	322	0	0	0	62 (19)
Plasmid pRK1-3	CP062807	125,309	60.2	132	0	0	0	54 (41)
Plasmid pRK1-4	CP062808	82,842	62.6	104	0	0	0	40 (38)
Plasmid pRK1-5	CP062809	81,787	62.3	81	0	0	0	14 (17)
Plasmid pRK1-6	CP062810	2,515	59.2	3	0	0	0	1 (33)
Total	NA^*b*^	8,942,610	65.0	8,060	21	7	67	1,082 (13)

a CDS, coding DNA sequences.

b NA, not applicable.

The key genes involved in chloroaromatic degradation, encoding chlorophenol monooxygenases (GenBank accession number QOT82435 and QOT82420), chlorohydroquinone 1,2-dioxygenase (QOT82419), and chlorocatechol 1,2-dioxygenases (QOT82433 and QOT82442), are located on plasmid pRK1-5 (CP062809). Additionally, *C. basilensis* DSM 11853^T^ has extensive catabolic potential, harboring nearly all major central pathways for aromatic compounds (15), including catechol 1,2-dioxygenase (QOT79538), catechol 2,3-dioxygenase (QOT80779), protocatechuate 3,4-dioxygenase (QOT80900 and QOT81306), homoprotocatechuate 2,3-dioxygenase (QOT78968), gentisate 1,2-dioxygenase (QOT81130), and homogentisate 1,2-dioxygenase (QOT81322), all of them located on chromosome 2 (CP062804), among other ring-cleavage enzymes.

This complete genome sequence represents a valuable taxonomic reference within the genus *Cupriavidus* and the family *Burkholderiaceae* and offers a genetic basis for elucidating the catabolic pathways for chloroaromatic compounds in this specialized bacterium.

### Data availability.

This complete genome sequence has been deposited in DDBJ/ENA/GenBank under the accession numbers CP062803, CP062804, CP062805, CP062806, CP062807, CP062808, CP062809, and CP062810. The versions described in this paper are the first versions. The Illumina and Oxford Nanopore raw sequence reads are available in the Sequence Read Archive under the accession numbers SRR12739612, SRR12739613, SRR12739614, and SRR12739615.
